# Supplementary material for: Expression of IDO1 and PD-L2 in Patients with Benign Lymphadenopathies and Association with Autoimmune Diseases
Source: Biomolecules. 2023 Jan 27;13(2):240. doi: 10.3390/biom13020240 (PMC9952948; doi:10.3390/biom13020240)
Supplement: Supplementary file 1 [file biomolecules-13-00240-s001.zip › biomolecules-2096431-supplementary.pdf]

Supplementary Table S1. Patients with autoimmune diseases (n=22) described according to median proportions of IDO1+ and PD-L2+ cells.

Variable	Interfollicular IDO1+ cells ≥5%, n (%) n=15	Interfollicular IDO1+ cells <5%, n (%) n=6	P value *	Follicular IDO1+ cells ≥1% n=3	Follicular IDO1+ cells <1% n=15	P value**	Interfollicular PDL2+ cells ≥5%, n (%) n=13	Interfollicular PDL2+ cells <5%, n (%) n=8	P value***	Follicular PDL2+ cells ≥5% n=11	Follicular PDL2+ cells <5% n=7	P value****
**RA**			0.36			0.25			0.67			0.33
Yes	6 (40)	4 (67)		0 (0)	7 (47)		5 (38)	4 (50)		3 (27)	4 (57)	
No	9 (60)	2 (33)		3 (100)	8 (53)		8 (62)	4 (50)		8 (73)	3 (43)	
**RA and/or SLE**			0.66			0.21			0.66			0.99
Yes	8 (53)	4 (67)		0 (0)	9 (60)		6 (46)	5 (63)		5 (45)	4 (57)	
No	7 (47)	2 (33)		3 (100)	6 (40)		7 (54)	3 (38)		6 (55)	3 (43)	
**RA and/or Sjögren's syndrome**			0.64			0.22			0.99			0.14
Yes	7 (47)	4 (67)		0 (0)	8 (53)		6 (46)	4 (50)		3 (27)	5 (71)	
No	8 (53)	2 (33)		3 (100)	7 (47)		7 (54)	4 (50)		8 (73)	2 (29)	
**RA, SLE and/or Sjögren's syndrome**			0.99			0.07			0.99			0.37
Yes	9 (60)	4 (67)		0 (0)	10 (67)		7 (54)	5 (63)		5 (45)	5 (71)	
No	6 (40)	2 (33)		3 (100)	5 (33)		6 (46)	3 (38)		6 (55)	2 (29)	

IDO1=Indoleamine 2,3 dioxygenase; PD-L2=Programmed death ligand 2; RA=Rheumatoid arthritis; SLE=Systemic lupus erythematosus

*Comparing interfollicular IDO1+ cells ≥5% with interfollicular IDO1+ cells <5%

** Comparing follicular IDO1+ cells ≥1% with follicular IDO1+ cells <1%

***Comparing interfollicular PD-L2+ cells ≥5% with interfollicular PD-L2+ cells <5%

**** Comparing follicular PD-L2+ cells ≥5% with follicular PD-L2+ cells <5%

Supplementary Table S2. Correlation of interfollicular and follicular cells in patients with autoimmune diseases. Spearman rho correlation coefficient and P-value.

	Follicular IDO1+ cells	Interfollicular PD-L2+ cells	Follicular PD-L2+ cells	Interfollicular PD-1+ cells	Follicular PD-1+ cells	Interfollicular PD-L1+ cells	Follicular PD-L1+ cells	Interfollicular EBV+ cells	Follicular EBV+ cells
**Interfollicular IDO1+ cells**	0.35<br>0.16	0.30<br>0.20	-0.23<br>0.37	-0.25<br>0.28	0.02<br>0.92	**0.69**<br>**<0.001**	0.05<br>0.83	**0.56**<br>**0.008**	-0.04<br>0.86
**Follicular IDO1+ cells**		0.04<br>0.89	0.07<br>0.78	0.04<br>0.89	0.18<br>0.47	**0.52**<br>**0.03**	0.22<br>0.37	**0.54**<br>**0.02**	**0.61**<br>**0.007**
**Interfollicular PD-L2+ cells**			-0.30<br>0.22	-0.09<br>0.70	-0.17<br>0.48	0.18<br>0.44	-0.14<br>0.57	0.03<br>0.91	-0.20<br>0.41
**Follicular PD-L2+ cells**				0.44<br>0.07	0.41<br>0.09	0.09<br>0.71	**0.53**<br>**0.02**	0.05<br>0.84	0.14<br>0.57

IDO1=Indoleamine 2,3 dioxygenase; PD-L2=Programmed death ligand 2; PD-1=Programmed death receptor 1; EBV=Epstein-Barr Virus

Supplementary Table S3. Correlation of interfollicular and follicular cells in patients without autoimmune diseases. Spearman rho correlation coefficient and P-value.

	Follicular IDO1+ cells	Interfollicular PD-L2+ cells	Follicular PD-L2+ cells	Interfollicular PD-1+ cells	Follicular PD-1+ cells	Interfollicular PD-L1+ cells	Follicular PD-L1+ cells	Interfollicular EBV+ cells	Follicular EBV+ cells
**Interfollicular IDO1+ cells**	**0.41**<br>**0.002**	-0.11<br>0.42	0.09<br>0.55	-0.13<br>0.34	-0.21<br>0.14	0.20<br>0.14	0.19<br>0.19	0.24<br>0.07	0.07<br>0.62
**Follicular IDO1+ cells**		-0.11<br>0.42	0.19<br>0.17	-0.13<br>0.36	0.08<br>0.60	0.21<br>0.13	0.23<br>0.10	0.26<br>0.07	0.10<br>0.47
**Interfollicular PD-L2+ cells**			**0.36**<br>**0.01**	0.15<br>0.28	-0.03<br>0.82	0.05<br>0.70	0.03<br>0.86	-0.15<br>0.25	0.06<br>0.67
**Follicular PD-L2+ cells**				0.14<br>0.31	0.05<br>0.75	0.09<br>0.52	**0.36**<br>**0.01**	0.21<br>0.14	0.24<br>0.08

IDO1=Indoleamine 2,3 dioxygenase; PD-L2=Programmed death ligand 2; PD-1=Programmed death receptor 1; EBV=Epstein-Barr Virus
